# Clinical Reasoning and Knowledge Assessment of Rheumatology Residents Compared to AI Models: A Pilot Study

**DOI:** 10.3390/jcm13237405

**Published:** 2024-12-05

**Authors:** Esra Kayacan Erdoğan, Hakan Babaoğlu

**Affiliations:** Department of Rheumatology, Division of Internal Medicine, Ankara Bilkent City Hospital, Ankara 06800, Turkey; hakanbabaoglu@gmail.com

**Keywords:** artificial intelligence, large language model, arthritis, rheumatology

## Abstract

**Background:** The integration of artificial intelligence (AI) in medicine has progressed from rule-based systems to advanced models and is showing potential in clinical decision-making. In this study, the psychological impact of AI collaboration in clinical practice is assessed, highlighting its role as a support tool for medical residents. This study aimed to compare clinical decision-making approaches of junior rheumatology residents with both trained and untrained AI models in clinical reasoning, pre-diagnosis, first-line, and second-line management stages. **Methods:** Ten junior rheumatology residents and two GPT-4 models (trained and untrained) responded to 10 clinical cases, encompassing diagnostic and treatment challenges in inflammatory arthritis. The cases were evaluated using the Revised-IDEA (R-IDEA) scoring system and additional case management metrics. In addition to scoring clinical case performance, residents’ attitudes toward AI integration in clinical practice were assessed through a structured questionnaire, focusing on perceptions of AI’s potential after reviewing the trained GPT-4’s answers. **Results:** Trained GPT-4 outperformed residents across all stages, achieving significantly higher median R-IDEA scores and superior performance in pre-diagnosis, first-line, and second-line management phases. Residents expressed a positive attitude toward AI integration, with 60% favoring AI as a supportive tool in clinical practice, anticipating benefits in competence, fatigue, and burnout. **Conclusions:** Trained GPT-4 models outperform junior residents in clinical reasoning and management of rheumatology cases. Residents’ positive attitudes toward AI suggest its potential as a supportive tool to enhance confidence and reduce uncertainty in clinical practice. Trained GPT-4 may be used as a supplementary tool during the early years of residency.

## 1. Introduction

Artificial intelligence (AI) has revolutionized technology and science, with its transformative impact now extending significantly into medicine. Alan Turing’s 1950 research established the foundational concept of electronic decision-making systems [1]. Over time, Turing’s model advanced into diverse subfields including machine learning, deep learning, natural language processing, and computer vision [2,3]. While the use of artificial intelligence has progressed faster in engineering disciplines, it has now found significant applications in medicine, mirroring developments across various scientific fields.

In medicine, AI is utilized across multiple domains, such as data collection and management, imaging methods, and treatment recommendations [4,5]. One of the earliest AI applications in healthcare was MYCIN, a backward chaining artificial intelligence system developed in the early 1970s. MYCIN was able to provide a list of potential bacterial pathogens according to patient information and recommend an appropriate treatment [6]. In a similar period, a consultation system for glaucoma was developed using the CASNET model and was officially demonstrated at the meeting of the Academy of Ophthalmology in 1976. This model was able to apply knowledge about a particular disease to individual patients and advise physicians on management [7]. This was followed by the development of Dxplain in 1986, a tool designed to assist with differential diagnosis [8]. These early tools laid the groundwork for today’s sophisticated AI systems, highlighting the potential of automated reasoning in clinical decision-making.

As AI evolved, it transitioned into what is now commonly known as deep learning, which introduced neural networks capable of self-learning, enabling AI to make decisions akin to human reasoning. A landmark moment in the use of AI in medicine occurred in 2017, with the Food and Drug Administration’s approval of Artery, the first deep learning-based application. Artery’s initial deployment, CardioAI, focused on analyzing cardiac magnetic resonance imaging, followed by extensions into liver, lung, and musculoskeletal imaging [4].

Recent advancements in deep learning have significantly contributed to the development of large language models (LLMs). These models, trained on extensive datasets, have the capability to learn and interpret complex semantic relationships. LLM-based models, which can generate human-like responses and facilitate communication with users, have now become widely used in everyday applications. With their ability to synthesize complex semantic relationships, LLMs have found applications in clinical practice in medicine, particularly in aiding decision-making under uncertainty [9]. Notably, studies have shown that AI models can perform well in medical assessments such as the United States Medical Licensing Examination (USMLE), which measures not only cognitive skills and medical knowledge in medicine, but also the navigation of different scenarios, patient safety, and professional legal and ethical values [10,11]. Although LLMs have demonstrated competence in diagnostics and theoretical knowledge assessments, their effectiveness in real-world outpatient settings, particularly in clinical reasoning and patient management, requires further exploration.

In addition to evaluating theoretical knowledge and clinical application proficiency through board exams [10,11,12], recent research has explored the practical use of LLMs in clinical settings. Many of these studies focus on assessing diagnostic capabilities, either through retrospective analyses of real-life clinical cases or comparisons with clinicians using constructed scenarios. For instance, in a study involving 36 dermatology cases, an LLM demonstrated diagnostic accuracy comparable to non-specialists but performed inferiorly to dermatologists [13]. Similarly, in a comparative study involving an LLM-based AI model and internal medicine residents, the AI demonstrated superior capabilities in processing and reasoning through clinical information [14].

LLMs have also been assessed for their treatment recommendations. In a pilot study evaluating clinical MRI reports for orthopedic disorders, LLMs provided largely accurate suggestions for knee and shoulder conditions but often lacked specificity due to limited contextual awareness, making their responses less practical for real-world use [15]. Another study compared LLMs’ and neurologists’ responses in managing Alzheimer’s patients. While neurologists found the AI-generated recommendations adequate, caregivers perceived no significant difference between the two [16]. This suggests a promising role for AI in assisting clinical decision-making, potentially alleviating the stress and burnout often experienced by residents, especially in situations of diagnostic uncertainty [17].

As the integration of AI into clinical practice continues to evolve, its potential to enhance clinical reasoning and support decision-making has emerged as a focal point of research. While studies have highlighted AI’s capabilities in either diagnostics or treatment recommendations, a comprehensive evaluation encompassing diagnostics, clinical reasoning, treatment planning, and its potential psychological benefits remains underexplored. Addressing these gaps is essential to understanding AI’s role not merely as a diagnostic tool but as a holistic support system in medical education and clinical practice.

This study aims to evaluate and compare the clinical reasoning and decision-making capabilities of junior rheumatology residents with those of trained and untrained LLM-based AI models, focusing on clinical reasoning, pre-diagnosis, and treatment. Additionally, the potential psychological impact of AI collaboration during clinical practice is evaluated, offering insights into AI’s role as a supportive tool for residents.

## 2. Materials and Methods

We performed this study at three academic medical centers in Türkiye: Hacettepe University, Adana Cukurova University, and Bilkent City Hospital. Participants were recruited from a rheumatology residency program. Any rheumatology trainee, below 2 years of education, was eligible for study inclusion.

We developed 10 clinical cases, written by practicing rheumatologists, focusing on common outpatient care problems including approaches to arthritis, differential diagnosis of arthritis and back pain, and management of Rheumatoid arthritis and Axial Spondyloarthritis. Each case has sections for the review of systems, physical examination, diagnostic testing, differential diagnosis, treatment, treatment response or adverse effect, treatment modification, and follow-up.

We developed a survey to evaluate participant performance. The survey contained no demographic data, and participants were advised to use nicknames as their IDs. Once a response was submitted, participants were unable to modify their answers. Each case included approximately 30 open-ended questions, requiring participants to provide their responses in text form. Identical survey instructions were used for junior rheumatologists and GPT-4’s (Supplementary Table S1), both of which provided responses to all 10 cases (Supplementary Figure S1).

The GPT-4 model was trained using a comprehensive set of materials to improve its clinical decision-making in the context of inflammatory arthritis and back pain. This training included relevant medical algorithms and up-to-date clinical guidelines.

Each case included questions aimed at evaluating the R-IDEA score [18]. The R-IDEA assessment tool uses a 10-point scale to assess four essential components of clinical reasoning documentation: interpretive summary, differential diagnosis, justification of the primary diagnosis, and explanation of alternative diagnoses. The scoring is divided into a range of 0–4 for problem representation and 0–2 for each of the other three categories. R-IDEA scores were also binarized as low (<7) or high. The R-IDEA tool has strong interrater reliability, a predictive capability for educational outcomes in real-world settings, and may track the progression from novice to expert [18].

In addition to the R-IDEA evaluation, open-ended questions were used to assess participants’ clinical management skills. These questions covered both fundamental and intermediate aspects of case management, such as diagnosis, treatment response, and management of adverse effects, encouraging diverse and detailed responses. Scores were categorized into three phases.

Pre-diagnostic phase: questions up to the diagnostic stage.

First-line management: questions related to initial treatment and management after diagnosis.

Second-line management: questions addressing treatment failure, side effects, comorbidities, and subsequent modifications.

After completing the initial survey, participants reviewed the trained GPT-4’s responses and completed a follow-up survey to evaluate their perceptions of the impact of using trained GPT-4 in daily practice. The survey sought to assess whether the AI system could help reduce fatigue and burnout, while also enhancing learning experiences and supporting professional development.

For data scoring, 2 scorers (BA and HEK, both attending rheumatologist) were blinded to the hypothesis of the study, respondent type (GPT-4, trained GPT-4, rheumatology residents), and the training levels of participants. The R-IDEA questions were scored by raters based on the corresponding article [18] (Supplementary Tables S2 and S3). The additional questions were also evaluated by the raters using a 5-point Likert scale (Strongly Disagree, Disagree, Neutral, Agree, Strongly Agree). The scoring involved taking the average of the two scorers’ ratings, with a review process in place in the event of significant deviations (>1) between the scorers’ assessments (Supplementary Figure S2).

The average scores of each answer were used. Statistical analyses were conducted using Jamovi Project (2022, Jamovi Version 2.3, Computer Software) [19]. Descriptive statistics were calculated using the median (25–75th) for continuous outcomes and the percentage for binary outcomes. Each participant had at least 10 R-IDEA scores, all of which were evaluated as separate instances. The numeric Likert scale responses from the non-R-IDEA case management questions were divided into three stages for each case: pre-diagnostic phase, first-line management, and second-line management. R-IDEA scores were binarized as low and high. These scores from R-IDEA, pre-diagnostic, first-line, and second-line management were compared between residents and GPT-4 models. In our study, case vignettes were created by us and did not involve real patient data. Rheumatology residents responded to these case vignettes without sharing any personal data, and their responses were then compared to those generated by the AI model. As such, this research did not involve any real patient information or direct interaction with humans. Therefore, the study is exempt from ethics committee approval.

## 3. Results

**Figure 1:**
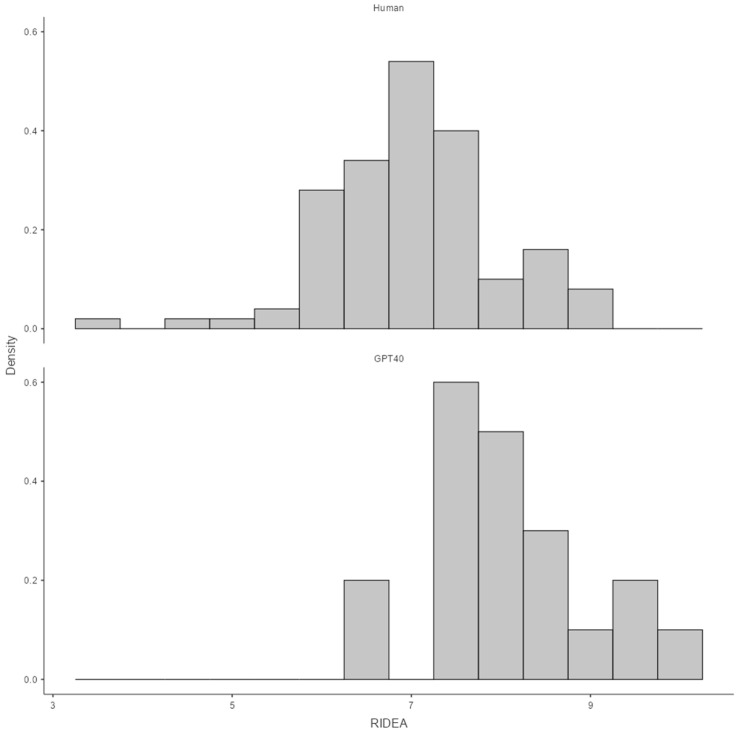
Distribution of R-IDEA scores across residents and GPT-4 Models. R-IDEA: Revised IDEA.

The sample included responses from 10 residents and two GPT-4 models (one trained and one untrained), each answering questions regarding the pre diagnosis, R-IDEA scores, first-line treatment, and second-line treatment of ten cases. The analysis focused on comparing the performance of residents with both trained and untrained GPT-4 models. Median (25th–75th) R-IDEA scores were 8.0 (7.5–8.5) for GPT-4 and 7.0 (6.5–7.5) for residents. GPT-4 had significantly higher R-IDEA scores than residents (*p* < 0.01) (Figure 1).

Specifically, the trained GPT-4 model had a median score of 8.5 (8.0–9.5), while the untrained GPT-4 model scored 7.5 (7.4–7.5). Residents had a median R-IDEA score of 7.0 (6.5–7.5). Statistical analysis revealed that the trained GPT-4 significantly outperformed the residents and untrained GPT-4 model in R-IDEA scores (*p* < 0.001). However, further analyses revealed no significant difference between the untrained GPT-4 model and residents (*p* = 0.08) (Table 1).

When R-IDEA scores were categorized as “high” (≥7) or “low” (<7), the trained GPT-4 demonstrated a superior performance, with 100% of its scores falling into the “high” category, compared to 64% for residents (*p* = 0.02). The untrained GPT-4 also outperformed residents in this categorical analysis, with 80% of its scores classified as “high,” though this difference was not statistically significant (*p* = 0.31).

**Figure 2:**
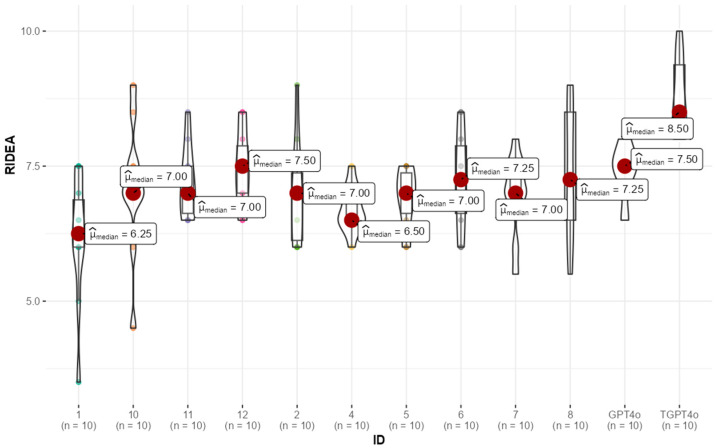
Illustrates the distribution of R-IDEA scores across all participants using box violin plots. R-IDEA: Revised IDEA.

The distribution of participant R-IDEA scores, visualized via box-violin plots (Figure 2), highlights a clear pattern. Most residents achieved scores comparable to the untrained GPT-4 model, while the trained GPT-4 model exhibited significantly higher performance. This suggests that while the untrained GPT-4 and residents performed similarly, the trained GPT-4 consistently outperformed each resident.

In the pre-diagnosis stage, both GPT-4 models performed better than the residents. The trained GPT-4 had a median pre-diagnosis score of 5.0 (4.8–5.0), significantly higher than the residents’ median score of 3.8 (3.3–4.1), with a *p*-value of less than 0.001. Similarly, the untrained GPT-4 scored 4.5 (4.5–4.8), also surpassing the residents’ performance (*p* < 0.001). However, there was a statistically significant difference between the trained and untrained GPTs (*p* = 0.007), highlighting the trained model’s advantage.

In terms of first-line treatment, the trained GPT-4 once again outperformed the residents, with a median score of 4.8 (4.5–4.9) compared to the residents’ median score of 4.0 (3.6–4.0) (*p* < 0.001). The untrained GPT-4 also had a higher median score of 4.1 (3.8–4.5) than the residents, but this difference was not statistically significant (*p* = 0.14). For second-line treatment, the trend continued, with the trained GPT-4 achieving a median score of 4.9 (4.5–5.0), significantly better than the residents’ median score of 4.0 (3.5–4.3) (*p* < 0.001). The untrained GPT-4’s median score was 4.5 (4.4–4.7), also higher than the residents’, with statistical significance (*p* < 0.001). Trained GPT was found to be statistically better than untrained GPT-4 in terms of first-line management and there was a trend towards this in second-line management as well (*p* = 0.008 and *p* = 0.028, respectively). The clear distinction in performance is visualized in Supplementary Figure S4, where the box-violin plots show that most residents performed similarly to the untrained GPT-4 in first-line management, but that the trained GPT-4 demonstrated superior scores to each resident.

Following the initial study, a post-survey was conducted where the residents were presented with the responses generated by the trained GPT-4 model. The survey aimed to assess the residents’ perceptions of using a similar AI program in clinical practice.

The results indicated that 60% of the residents expressed a preference for having access to an AI program with similar capabilities to assist them in patient evaluations during clinical work. When asked if they would feel more competent in patient care when working with such a program, 60% responded affirmatively. Additionally, 60% of the residents believed that using the AI program could contribute to reducing their fatigue at the end of the day and that it could generally improve their working conditions. Moreover, 60% felt that the AI program could help alleviate the burnout associated with their working environment.

A notable 70% of the participants agreed that the AI-generated responses had contributed to their education, highlighting the potential educational benefits of incorporating AI tools in medical training.

## 4. Discussion

Our findings provide insightful evidence on the comparative performance of junior rheumatology residents and AI models (both trained and untrained) in clinical reasoning and knowledge. Using the R-IDEA tool and additional metrics, we demonstrated the superior performance of the trained GPT-4 model across all evaluation phases—clinical reasoning, pre-diagnosis, first-line management and second-line management—compared to junior residents. Notably, trained GPT-4’s greater consistency compared to residents and untrained models, suggesting its potential as a tool to support clinical decision-making and training. Also, residents gave positive comments regarding the potential for AI integration to boost competence and learning, reduce fatigue, and mitigate burnout. Unlike previous studies, our research provides a comprehensive evaluation of AI’s role across diagnostic processes, clinical reasoning, and treatment management. Additionally, it highlights the psychological benefits of AI integration for junior residents, including perceived reductions in fatigue and burnout, as well as improvements in confidence and learning. These findings position AI tools as valuable adjuncts for medical education and clinical practice, particularly in early-stage training.

The trained GPT-4 model significantly outperformed residents across all clinical reasoning and management phases, demonstrating its potential to enhance diagnostic accuracy and support clinical decision-making. Our finding partially aligns with existing literature; GPT-4 achieves diagnostic accuracy comparable to non-specialists but falls short when benchmarked against dermatologists [13]. Another study revealed that GPT models excelled in clinical reasoning, demonstrating diagnostic accuracy and correct decision-making comparable to residents and specialists, although were less effective in handling complex clinical reasoning compared to specialists [14]. A comparative study involving junior urology residents found that GPT models produced similar results to residents in urological cases [20]. The superior performance of GPT-4 in our study may stem from the comparison group being early residents rather than expert rheumatologists.

A study by Truhn et al. assessed GPT’s treatment recommendations based on MRI findings. Responses generated by GPT were evaluated using a Likert scale by two expert orthopedists [15]. The findings revealed that GPT’s recommendations were contemporary, detailed, and aligned with scientific evidence. Although this study did not employ an evaluation scale directly comparable to the R-IDEA, GPT’s reasoning capabilities were nonetheless regarded as robust. However, limitations such as reduced awareness of the patient’s overall condition, the potential for misjudging urgency, and nonspecific treatment recommendations led researchers to conclude that GPT serves better as a supportive tool for clinicians rather than a standalone alternative. Nonetheless, the demonstrated ability of GPT to provide a broad perspective in patient evaluations and strong reasoning capabilities supports our assertion that GPT models hold potential as an adjunct in routine clinical practice. These findings further affirm GPT’s value in assisting with comprehensive patient assessments, despite its limitations. Consequently, although GPT will not be accepted as a health professional on its own, it seems to be a technology that can be used especially to support residents [20].

The score distribution of residents’ responses varied widely across different cases, while both trained and untrained GPT-4’s maintained a narrower score range, reflecting greater consistency in performance (Figure 2, Supplementary Figures S3–S5). This suggests that AI may offer a more standardized approach to patient care, especially for residents who are still developing their specialty skills. Only trained AI models outperformed residents in the “first-line” but both AI models did in “second-line” management phases, suggesting an advantage of AI in addressing more complex scenarios such as treatment failure and comorbidities, likely due to the junior resident’s limited exposure to complex, treatment-resistant cases during early training. Although we cannot definitively determine the reasons, the willingness of junior residents to integrate AI into their practice may stem from their desire for support in situations where they feel uncertain or inadequate. Knowing they can rely on AI tools like GPT for guidance in challenging cases could explain their positive attitudes toward AI integration.

While AI models have become increasingly integral to data collection and imaging in medicine, their potential role in clinical decision support continues to expand. Our findings revealed significant differences between trained and untrained GPT-4 models in certain areas, highlighting the need for the further refinement of AI tools to ensure consistent performance across diverse clinical scenarios. This demonstrates that AI still has significant room for improvement and optimization, emphasizing the importance of developing AI systems as optimal clinical decision support and training tools. To achieve this, it is crucial to involve physicians in the development process, ensuring that these tools align with real-world clinical needs and facilitate meaningful integration into medical practice. Although GPT models may not match the experience and intuition of an experienced physician, they can serve as valuable support systems for junior residents, especially in early training stages, benefiting both junior residents and patients.

An important aspect of this study was the evaluation of residents’ perceptions of AI integration into their practice. Residents expressed an overwhelmingly positive response to AI integration, with 60% indicating a preference for using similar AI tools in clinical practice to boost competence, reduce fatigue, and mitigate burnout. Furthermore, 70% agreed that AI-generated responses enhanced their learning, underscoring the potential of AI models to support medical training and clinical reasoning development. Fatigue, depression, and burnout are prevalent among residents due to intense workloads and feelings of professional inadequacy. Literature shows that these factors negatively affect physician well-being, patient satisfaction, and safety [21,22]. A study of 123 pediatric residents in three U.S. hospitals found that 20% met criteria for depression and 74% for burnout, with medical errors being six times higher among depressed residents [23]. Uncertainty and stress, common in junior doctors due to limited experience, are associated with higher risks of burnout [17]. Integrating advanced AI models like GPT-4 into medical education and clinical practice could enhance patient care outcomes and may reduce uncertainty and burnout and support mental well-being among residents, as reflected in their expectations.

Despite the potential benefits, integrating AI into clinical practice raises critical ethical concerns. Transparency in AI decision-making and accountability for errors remain unresolved issues. Furthermore, biases in AI training datasets could disproportionately impact certain patient populations, leading to inequities in care. For junior residents, over-reliance on AI could inadvertently hinder the development of independent clinical judgment. Addressing these challenges will be essential for ensuring the responsible and equitable use of AI tools in medicine. Although some guidelines have been published, these have not yet been clarified in clinical practice [24]. Nonetheless, the potential benefits of AI as a supportive tool in medical education and training appear undeniable. By enhancing learning, providing consistent guidance, and supporting clinical decision-making, AI tools like GPT hold promise for shaping the future of medical education and practice.

There are a number of limitations to this study. First, the use of fictional cases, while providing a standardized framework for comparison, may not fully replicate the complexities of real-world clinical scenarios, such as patient interactions and dynamic decision-making. The observed psychological and educational benefits were based on residents’ perceptions. Additionally, the reliance on self-reported evaluations of fatigue and burnout, rather than standardized scales, may introduce subjective bias, warranting further research using objective metrics. Finally, the small sample size is another limitation.

## 5. Conclusions

In conclusion, our study demonstrates that trained GPT models outperform junior residents in clinical reasoning and case management across diagnostic, first-line, and second-line treatment phases. These findings highlight the potential of AI tools as clinical support systems, offering psychological benefits by reducing uncertainty and stress while enhancing confidence and learning among junior doctors. By augmenting medical education and training, AI tools like GPT-4 could improve early-stage residency experiences and patient care outcomes. However, trained GPT-4 should currently be considered only as a learning tool during the early years of residency, as its performance has not been tested in real-world scenarios and there are no established ethical guidelines to support its use in clinical practice. Future studies should validate these results using real-world scenarios, more respondents, and standardized tools, while also addressing ethical concerns to ensure the effective and responsible use of AI as a support tool in clinical settings.

## Figures and Tables

**Table 1:** Summary of R-IDEA, R-IDEA binary, pre-diagnosis, first-line, and second-line treatment scores.

Variable	Human (N = 100)	Trained GPT-4 (N = 10)	GPT-4 (N = 10)	Total (N = 120)	*p*-Value
**R-IDEA**					<0.001
Median (25–75th)	7.0 (6.5–7.5)	8.5 (8.1–9.4)	7.5 (7.5–7.5)	7.0 (6.5–7.5)	
Range	3.5–9.0	8.0–10.0	6.5–8.0	3.5–10.0	
R-IDEA * (cut of 7)					0.047
Low	36 (36.0%)	0 (0.0%)	2 (20.0%)	38 (31.7%)	
High	64 (64.0%)	10 (100.0%)	8 (80.0%)	82 (68.3%)	
**Pre-diagnosis**					<0.001
Median (25–75th)	3.8 (3.3–4.1)	5(4.8–5.0)	4.5 (4.5–4.7)	4.0 (3.5–4.3)	
Range	2.8–4.8	4.5–5.0	4.0–5.0	2.8–5.0	
**First-line**					<0.001
Median (25–75th)	4.0 (3.6–4.0)	4.8 (4.5–4.9)	4.1 (3.8–4.5)	4.0 (3.7–4.3)	
Range	1.5–4.8	4.3–5.0	3.4–5.0	1.5–5.0	
**Second-line**					<0.001
Median (25–75th)	4.0 (3.5–4.3)	4.9 (4.5–5.0)	4.5 (4.4–4.7)	4.0 (3.5–4.5)	
Range	1.0–5.0	4.1–5.0	4.0–4.7	1.0–5.0	

R-IDEA: Revised IDEA. * “high” (≥7) or “low” (<7).

## Data Availability

Data can be shared if necessary.

## Supplementary Materials

The following supporting information can be downloaded at: https://www.mdpi.com/article/10.3390/jcm13237405/s1, Table S1. Survey instruction given to respondents (Both physician and GPTs,); Table S2. Instructions for Scorers; Table S3. The Revised-IDEA Assessment Tool for Clinical Reasoning Documentation by Schaye and Colleagues; Figure S1. Data collection; Figure S2. Example of the scoring process by two evaluators for two different respondents; Figure S3. Illustrates the distribution of pre-diagnosis scores across all participants using box-violin plots; Figure S4. Illustrates the distribution of first-line management scores across all participants using box-violin plots; Figure S5. Illustrates the distribution of second-line management scores across all participants using box-violin plots.

## References

[1] Amisha, Malik P., Pathania M., Rathaur V.K. (2019). Overview of artificial intelligence in medicine. J. Fam. Med. Prim. Care.

[2] Kaul V., Enslin S., Gross S.A. (2020). History of artificial intelligence in medicine. Gastrointest. Endosc..

[3] Ito S., Mine Y., Urabe S., Yoshimi Y., Okazaki S., Sano M., Koizumi Y., Peng T.-Y., Kakimoto N., Murayama T. (2024). Prediction of a Cephalometric Parameter and Skeletal Patterns from Lateral Profile Photographs: A Retrospective Comparative Analysis of Regression Convolutional Neural Networks. J. Clin. Med..

[4] Crotti N. (2017). Startup Arterys wins FDA clearance for AI-assisted cardiac imaging system. MedCity News.

[5] Matsinhe C., Kagodora S.B., Mukheli T., Mokoena T.P., Malebati W.K., Moeng M.S., Luvhengo T.E. (2024). Machine Learning Algorithm-Aided Determination of Predictors of Mortality from Diabetic Foot Sepsis at a Regional Hospital in South Africa During the COVID-19 Pandemic. Medicina.

[6] Shortliffe E.H., Davis R., Axline S.G., Buchanan B.G., Green C.C., Cohen S.N. (1975). Computer-based consultations in clinical therapeutics: Explanation and rule acquisition capabilities of the MYCIN system. Comput. Biomed. Res..

[7] Weiss S., Kulikowski C.A., Safir A. (1978). Glaucoma consultation by computer. Comput. Biol. Med..

[8] Barnett G.O., Cimino J.J., Hupp J.A., Hoffer E.P. (1987). DXplain: An evolving diagnostic decision-support system. JAMA.

[9] Hadi M.U., Al Tashi Q., Qureshi R., Shah A., Muneer A., Irfan M., Zafar A., Shaikh M.B., Akhtar N., Hassan S.Z. (2023). A Survey on Large Language Models: Applications, Challenges, Limitations, and Practical Usage. TechRxiv.

[10] Brin D., Sorin V., Vaid A., Soroush A., Glicksberg B.S., Charney A.W., Nadkarni G., Klang E. (2023). Comparing ChatGPT and GPT-4 performance in USMLE soft skill assessments. Sci. Rep..

[11] Gilson A., Safranek C.W., Huang T., Socrates V., Chi L., Taylor R.A., Chartash D. (2023). How Does ChatGPT Perform on the United States Medical Licensing Examination (USMLE)? The Implications of Large Language Models for Medical Education and Knowledge Assessment. JMIR Med. Educ..

[12] Katz U., Cohen E., Shachar E., Somer J., Fink A., Morse E., Shreiber B., Wolf I. (2024). GPT versus Resident Physicians—A Benchmark Based on Official Board Scores. NEJM AI.

[13] Stoneham S., Livesey A., Cooper H., Mitchell C. (2024). ChatGPT versus clinician: Challenging the diagnostic capabilities of artificial intelligence in dermatology. Clin. Exp. Dermatol..

[14] Cabral S., Restrepo D., Kanjee Z., Wilson P., Crowe B., Abdulnour R.-E., Rodman A. (2024). Clinical Reasoning of a Generative Artificial Intelligence Model Compared With Physicians. JAMA Intern. Med..

[15] Truhn D., Weber C.D., Braun B.J., Bressem K., Kather J.N., Kuhl C., Nebelung S. (2023). A pilot study on the efficacy of GPT-4 in providing orthopedic treatment recommendations from MRI reports. Sci. Rep..

[16] Zeng J., Zou X., Li S., Tang Y., Teng S., Li H., Wang C., Wu Y., Zhang L., Zhong Y. (2024). Assessing the Role of the Generative Pretrained Transformer (GPT) in Alzheimer’s Disease Management: Comparative Study of Neurologist- and Artificial Intelligence-Generated Responses. J. Med. Internet Res..

[17] Simpkin A.L., Khan A., West D.C., Garcia B.M., Sectish T.C., Spector N.D., Landrigan C.P. (2018). Stress From Uncertainty and Resilience Among Depressed and Burned Out Residents: A Cross-Sectional Study. Acad. Pediatr..

[18] Schaye V., Miller L., Kudlowitz D., Chun J., Burk-Rafel J., Cocks P., Guzman B., Aphinyanaphongs Y., Marin M. (2022). Development of a Clinical Reasoning Documentation Assessment Tool for Resident and Fellow Admission Notes: A Shared Mental Model for Feedback. J. Gen. Intern. Med..

[19] The Jamovi Project [Computer Software]. https://www.jamovi.org.

[20] Xv Y., Peng C., Wei Z., Liao F., Xiao M. (2023). Can Chat-GPT a substitute for urological resident physician in diagnosing diseases?: A preliminary conclusion from an exploratory investigation. World J. Urol..

[21] Dewa C.S., Loong D., Bonato S., Thanh N.X., Jacobs P. (2014). How does burnout affect physician productivity? A systematic literature review. BMC Health Serv. Res..

[22] Mateen F.J., Dorji C. (2009). Health-care worker burnout and the mental health imperative. Lancet.

[23] Fahrenkopf A.M., Sectish T.C., Barger L.K., Sharek P.J., Lewin D., Chiang V.W., Edwards S., Wiedermann B.L., Landrigan C.P. (2008). Rates of medication errors among depressed and burnt out residents: Prospective cohort study. BMJ.

[24] Ning Y., Liu X., Collins G.S., Moons K.G.M., McCradden M., Ting D.S.W., Ong J.C.L., Goldstein B.A., Wagner S.K., Keane P.A. (2024). An ethics assessment tool for artificial intelligence implementation in healthcare: CARE-AI. Nat. Med..

