# Supplementary material for: Clinical Reasoning and Knowledge Assessment of Rheumatology Residents Compared to AI Models: A Pilot Study
Source: J Clin Med. 2024 Dec 5;13(23):7405. doi: 10.3390/jcm13237405 (PMC11642710; doi:10.3390/jcm13237405)
Supplement: Supplementary file 1 [file jcm-13-07405-s001.zip › jcm-3307396-supplementary.pdf]

Supplementary Content

Supplementary Table S1. Survey instruction given to respondents (Both physician and GPTs, translated to English)

Supplementary Figure S1. Data collection (Translated to English)

Supplementary Table S2. Instructions for Scorers (Translated to English)

Supplementary Table S3. The Revised-IDEA Assessment Tool for Clinical Reasoning Documentation by Schaye and Colleagues

Supplementary Figure S2. Example of the scoring process by two evaluators (HEK and BA) for two different respondents

Supplementary Figure S3. Illustrates the distribution of pre-diagnosis scores across all participants using box-violin plots

Supplementary Figure S4. Illustrates the distribution of first-line management scores across all participants using box-violin plots

Supplementary Figure S5. Illustrates the distribution of second-line management scores across all participants using box-violin plots

**Supplementary Table S1.** Survey instruction given to respondents (Both physician and GPTs, translated to English)
You are a rheumatologist conducting outpatient clinic sessions. The cases presented to you are fictional but should be treated as though they involve real patients under your care. Each case will unfold in multiple sections, and your responses should reflect your clinical expertise as if you are actively managing the patient.<br>Each case will include the following sections:<br>1. **Prediagnosis:** You will evaluate the initial presentation of the patient, interpreting symptoms, medical history, and you will identify and request appropriate laboratory tests, imaging studies, and other diagnostic techniques necessary to narrow down the list of potential conditions.<br>2. **Clinical Reasoning:** You will explain your thought process in detail, justifying your differential diagnosis and guiding the next steps for diagnostic testing or clinical assessment.<br>3. **First-Line Management:** Based on the initial diagnosis, you will provide a detailed plan for first-line treatment, including medications, lifestyle advice, and monitoring strategies.<br>4. **Second-Line Management:** If first-line treatments are insufficient or additional complexities arise, you will develop a second-line management plan, incorporating advanced treatments, referrals, or other interventions as needed.<br>For each section, there will be open-ended questions related to the patient's case as it progresses. You must respond as if you are managing the patient in real life, with professional clinical reasoning, empathy, and evidence-based decision-making.<br>You will not be able to return to previous sections once the case moves forward, so treat each response as definitive. Each question demands a thorough, thoughtful answer that reflects the depth of your medical knowledge and patient care approach.

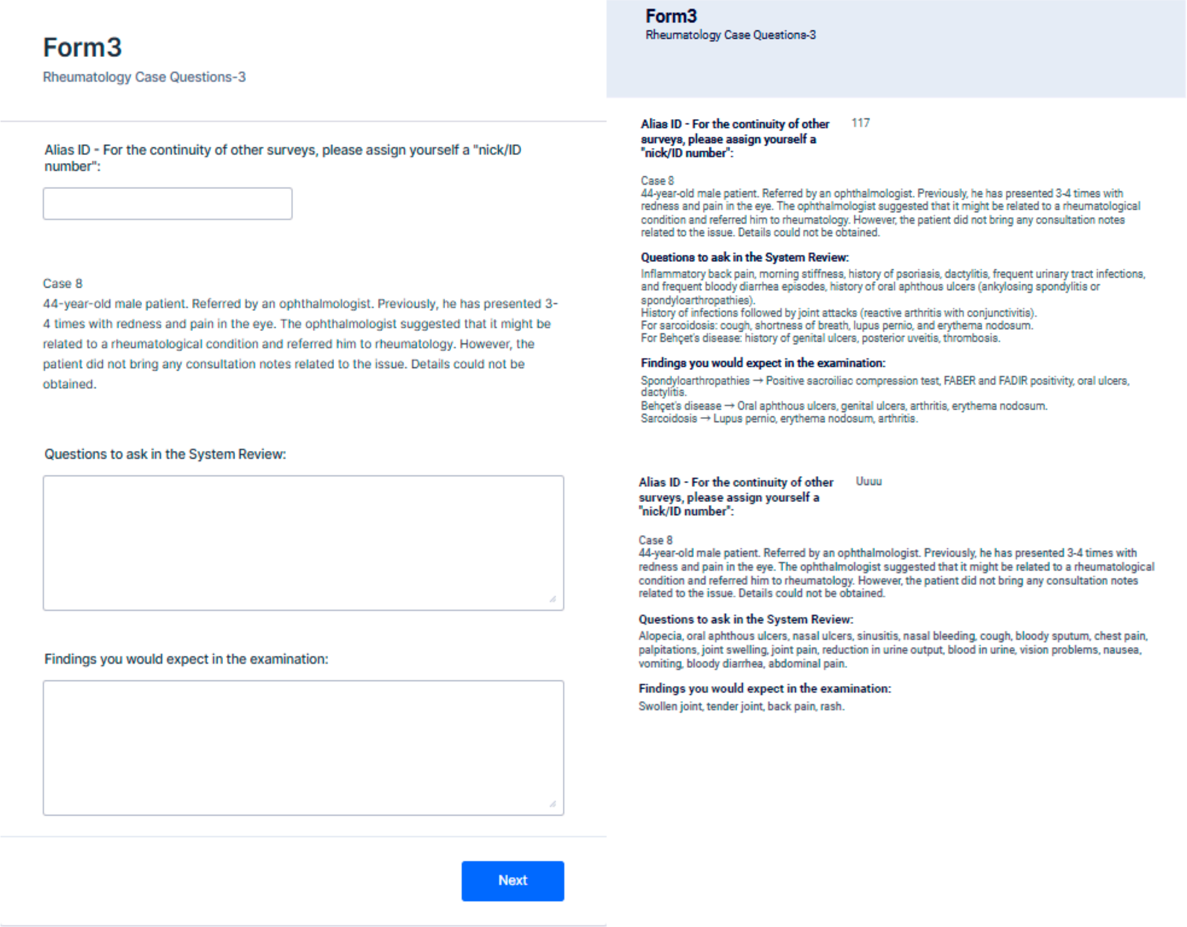

Supplementary Figure S1. Example of Data collection and two Responses, note that there is no back button in Survey (Translated to English)

**Supplementary Table S2.** Instructions for Scorers (Translated to English)
In the study, the answers given by 12 different participants to 10 cases will be shared with you for evaluation.<br>In the evaluation of each case, the questions prepared according to the standard RIDEA evaluation (such as key risk factors, differential diagnosis, etc.) will be made according to the RIDEA scale. The variables to be filled in with RIDEA scoring are labeled as Case1 RIDEA IA; Case1 RIDEA Ib, Case1 RIDEA Ic, Case1 RIDEA Id, Case1 RIDEA D, Case1 RIDEA E, Case1 RIDEA A, separately for each case.<br>There are other response labeled as Case1 Prediagnosis1, Case1 Prediagnosis2, Case 2 First line management1 etc. will be scored according to the Likert scale.<br>For the Likert scale, you are expected to evaluate as follows<br>1. I strongly disagree<br>2. I strongly disagree<br>3. Partially agree<br>4. I agree<br>5. Strongly agree<br><br>Detailed information on R-IDEA scales can be found below.

Supplementary Table S3. The Revised-IDEA Assessment Tool

Domain	Description	Assessment	Points
I - Interpretive Summary	Provides a concise summary statement that uses semantic vocabulary to highlight the most important elements from history, exam, and testing and to interpret and represent the patient's main problem(s). The presence or absence of the following features is assessed:<br>a) Key risk factors;<br>b) Chief complaint;<br>c) Illness time course; and<br>d) Use of semantic qualifiers (e.g. monoarticular vs polyarticular) or unified medical concepts (e.g. volume overload, cardiovascular risk factors).<br><br>NB: Some problems have an implied time course (e.g. syncope, seizure)	No features present	0
		1 feature present	2
		2 features present	2
		3 features present	3
		4 features present	4
D – Differential Diagnosis	Offers more than one relevant diagnostic possibility, committing to what is most likely and considering what is less likely or unlikely yet important to consider for the main chief complaint. If the chief complaint is a diagnosis or syndrome (e.g. acute on chronic systolic heart failure) then differential to rate may be around the differential for that exacerbation (e.g. medication non-compliance vs. arrhythmia).	No differantial	0
		Differential is implicitly stated, given as a diagnostic category (e.g. "cardiac"), OR implicitly prioritized	1
		Differential is explicitly stated AND explicitly prioritized	2
E – Explanation of Lead Diagnosis	Explains the reasoning behind the lead diagnosis, including the epidemiology and key features and how these compare with the patient's presentation. If objective data points are not clearly linked to the lead diagnosis or alternative diagnosis, then only designate points to lead OR alternative diagnosis and NOT both	No explanation	0
		1 objective data point in explanation of lead diagnosis	1
		≥2 objective data points in explanation of lead diagnosis	2

Domain	Description	Assessment	Points
A – Alternative Diagnosis Explained	Explains the reasoning behind alternative diagnoses, including the epidemiology and key features and how these compare with the patient's presentation and alternative diagnosis. If objective data points are not clearly linked to the lead diagnosis or alternative diagnosis, then only designate points to lead OR alternative diagnosis and NOT both	No explanation for any alternative diagnosis	0
		1 objective data point in explanation of at least one alternative diagnosis	1
		≥2 objective data points in explanation of at least one alternative diagnosis	2
Revised-IDEA Score	Overall evaluation of demonstration of clinical reasoning in the assessment section of admission notes	Sum of I + D + E + A points (score ≥6 indicates high-quality clinical reasoning documentation)	0-10

(Schaye V, Miller L, Kudlowitz D, et al. Development of a Clinical Reasoning Documentation Assessment Tool for Resident and Fellow Admission Notes: a Shared Mental Model for Feedback. J Gen Intern Med. 2022;37(3):507-512. doi:10.1007/s11606-021-06805-6)

	A	B	C	D	E	F	G	H	I	J	K	L
1	Scorer	Nickname	Case1 Prediagnosis1	Case1 Prediagnosis2	Case1 RIDEA Ia	Case 1 RIDEA Ib	Case 1 RIDEA Ic	Case 1 RIDEA Id	Case 1 RIDEA D	Case1 RIDEA A	Case1 RIDEA'E	Case1 Firstline 1
2	HEK	1	4	5	1	1	1	0	2	1	2	5
3	BA	1	5	5	1	1	1	0	2	1	2	4
4	HEK	19	4	5	1	1	1	1	1	0	1	5
5	BA	19	5	5	1	1	1	1	1	0	1	4

Supplementary Figure S2. Example of the scoring process by two evaluators (HEK and BA) for two different respondents.

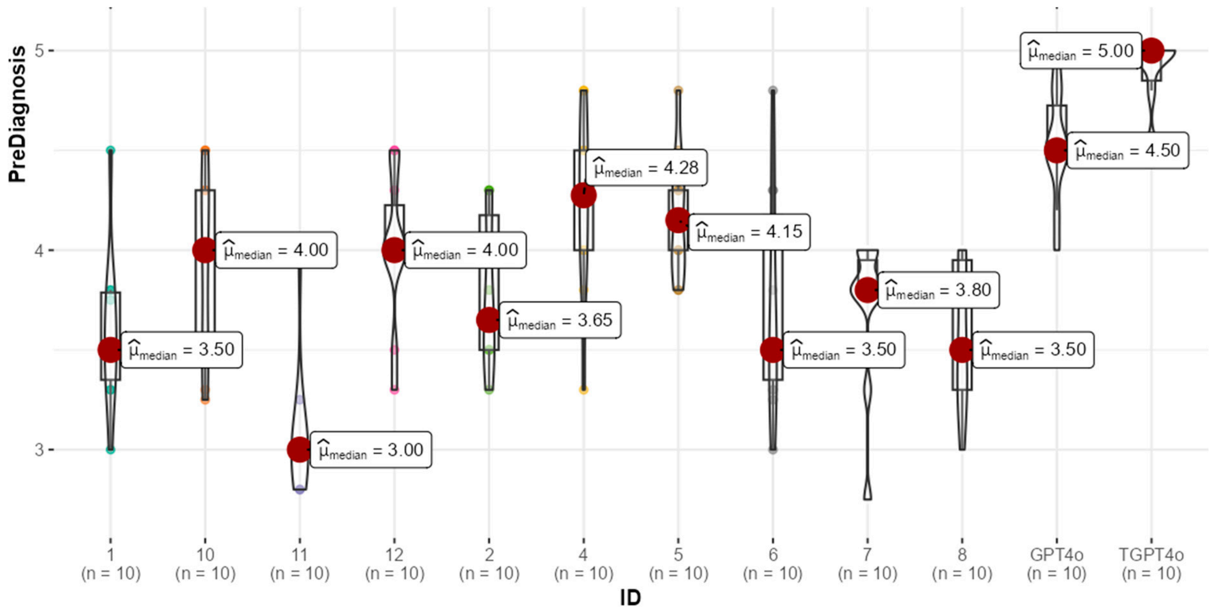

Supplementary Figure S3. Illustrates the distribution of pre-diagnosis scores across all participants using box-violin plots

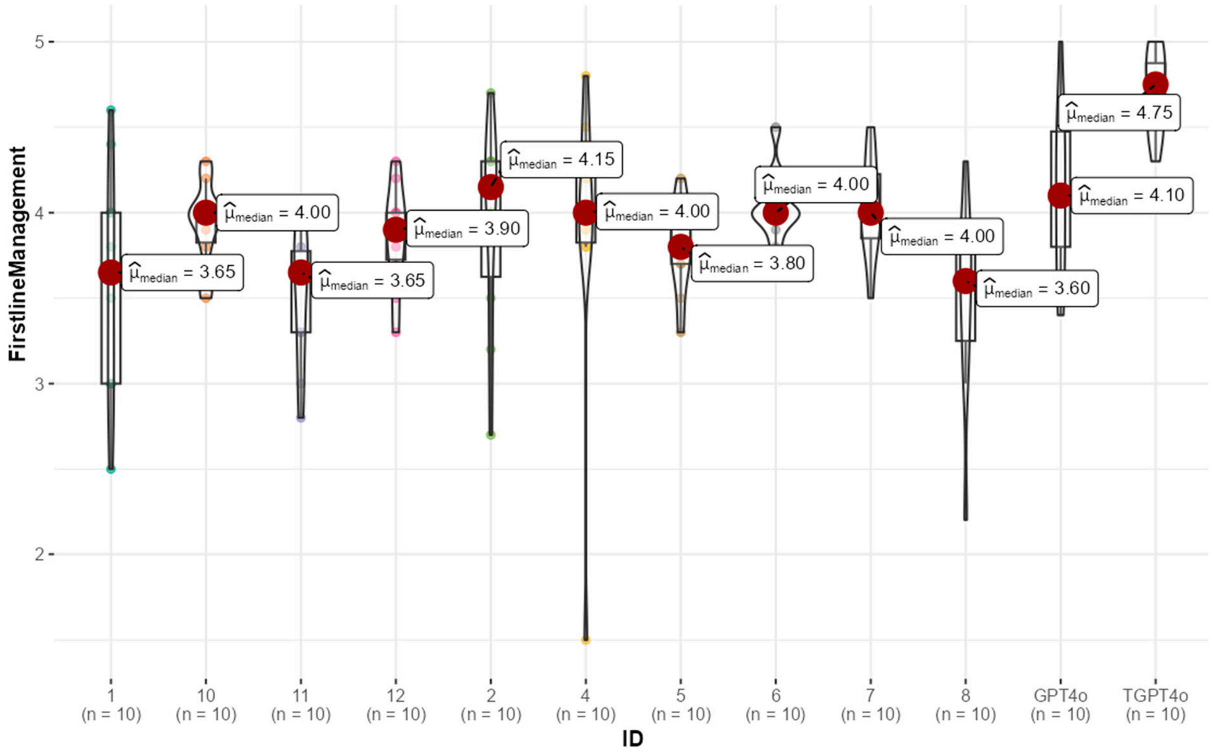

Supplementary Figure S4. Illustrates the distribution of first-line management scores across all participants using box-violin plots

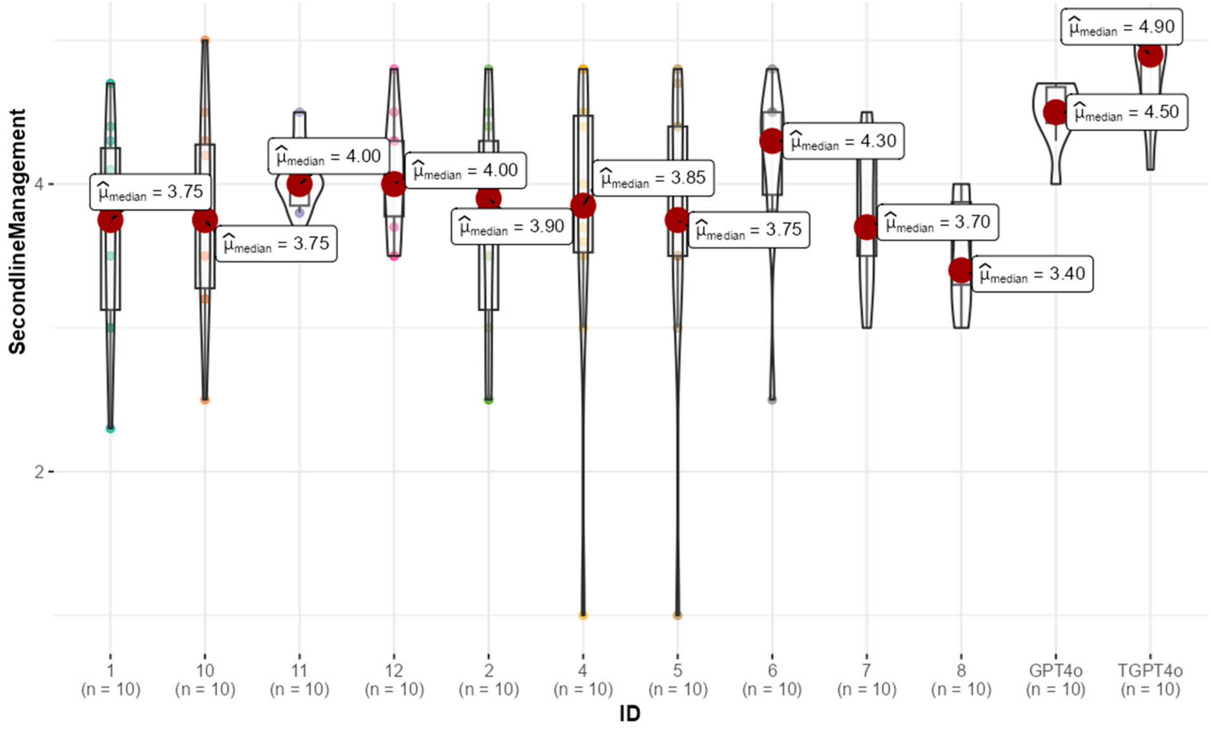

Supplementary Figure S5. Illustrates the distribution of second-line management scores across all participants using box-violin plots
